# Debate: COVID‐19 to the under 19 – a Singapore school mental health response

**DOI:** 10.1111/camh.12426

**Published:** 2020-10-13

**Authors:** Vidhya Renjan, Daniel S.S. Fung

**Affiliations:** ^1^ Department of Developmental Psychiatry Institute of Mental Health Singapore City Singapore

## Abstract

The 2019 novel coronavirus (COVID‐19) pandemic causes much disruption globally on sociopolitical, economic and healthcare fronts. While much of the impact has focused on the epidemiology and medical management of the pandemic, more need to be focused on the mental health impact of COVID‐19. This article describes the impact of COVID‐19 pandemic on Singapore’s schools and the response and adaptation of the school community mental health services. Singapore’s response is one of balancing the needs of the population and demands in this crisis, with the utilization of technology and outbreak and support control measures. Further consideration needs to be afforded to increase capacity of the school and mental health services to support youth and tapping on technological innovations.

The 2019 novel coronavirus (COVID‐19) pandemic poses a great challenge globally on economic, sociopolitical and healthcare fronts. Given the novelty and rate of transmission of COVID‐19, and the unpreparedness of many nations, much of the medical community’s efforts are focused on the epidemiology and medical management of COVID‐19, with less on the adverse impact on the mental health of individuals (Shah et al., 2020).

The COVID‐19 pandemic has resulted in loss of jobs, feelings of loneliness due to isolation and increased stress because of uncertainty and changes. It also profoundly affects children and adolescents, with social distancing and reduced outdoor activities including school closures in many countries. The United Nations currently estimates that nearly 1.6 billion students across more than 190 countries, more than 90% of learners globally, are affected. Due to school closures, many families have to balance the needs of homeschooling and parental work responsibilities, with reduced external caregiving support. As school routines help many students cope, its disruption has resulted in increased distress, mental health problems and child protection concerns during the pandemic (Fegert, Vitiello, Plener, & Clemens, 2020). The evolving pandemic also created additional strain on mental healthcare systems, which also needed to protect its patients and providers from infection across the globe, including Singapore (Poremski et al., 2020).

This article seeks to describe the impact of COVID‐19 pandemic on Singapore’s schools and the response and adaptation of the school community mental health services.

Singapore is a small island state situated within South‐East Asia with approximately 813,000 youth below the age of 20 years (Singapore Department of Statistics, 2019). The prevalence of emotional problems amongst primary school students is comparable to other developed nations at 12.5% (Woo et al., 2004). Mental disorders accounted for the largest portion of disease burden, about one‐third of disability‐adjusted life years (DALYs), in children aged below 15 years, and approximately 19% DALYs amongst those aged 15 and 34 years (Ministry of Health, 2014). Suicide is one of the leading causes of death amongst those aged 10–29 years, and the suicide rate was 5.7 per 100,000 and closely correlated with psychosocial stressors, including academic stress (Loh, Tai, Ng, Chia, & Chia, 2012).

To cope with increasing demands for mental health services amongst youth, the child and adolescent psychiatric service at the Institute of Mental Health (IMH), Singapore’s only psychiatric hospital, has had to evolve and adapt to changing needs to serve the population. The traditional model of care in outpatient and inpatient settings is unsustainable due to rapid growing demands and costs. In the past decade, there has been a shift to deinstitutionalize mental health services and establish a tiered‐care approach, particularly community‐based mental health services (Paxton, Shrubb, Griffiths, Cameron, & Maunder, 2000).

Thus, the Response, Early Intervention and Assessment in Community Mental Health (REACH) service was conceived in 2007 to support students with mental health issues. The main objectives of REACH are as follows: (a) to improve mental health of youth via early assessment and intervention; (b) to build capacity of schools and community partners to detect and manage mental health problems through training and support; and (c) to build a mental health support network for youths in the community.

REACH teams are regionally located to serve the regional school zones of Singapore. Each multidisciplinary team of psychiatrists, psychiatric nurses, psychologists, medical social workers and occupational therapists is linked to a regional hospital system to ensure continuity of care, networked with community social service agencies and primary care physicians. Details of REACH services and their outcomes are provided elsewhere (Lim, Loh, Renjan, Tan, & Fung, 2017).

The COVID‐19 pandemic poses many challenges to the usual operations of Singapore’s schools and REACH services. For example, during the stay‐at‐home order, or ‘circuit‐breaker’ period, which lasted approximately two months, schools closed. Singapore’s Ministry of Education (MOE) implemented full home‐based learning (HBL). Only students whose caregivers are working in essential services and those without adequate home support returned to school for lessons under enhanced safe management measures. All other students engaged in lessons online. During this period, school counsellors continued to support students who required additional support via telephone contact, on‐line sessions, or face‐to‐face engagement. School counsellors continued to refer students who required specialized support to REACH and other mental health professionals. When the ‘circuit‐breaker’ was extended, the mid‐year vacations were brought forward a month. Post‐‘circuit breaker’, students taking national examinations were scheduled to return to school for lessons earlier. Other students were on a weekly rotation schedule alternating between physical classes and online ones. A month after ‘circuit breaker’, all students returned to school with stringent hygiene and safe management measures, in line with the easing of national level restrictions in Singapore.

For REACH, the pandemic meant adapting to the evolving situation promptly to ensure continued services and support to students. Figure 1 depicts an increase in new referrals across child and adolescent mental health services at IMH at the height of the pandemic and decline when the ‘circuit breaker’ began.

**Figure 1:**
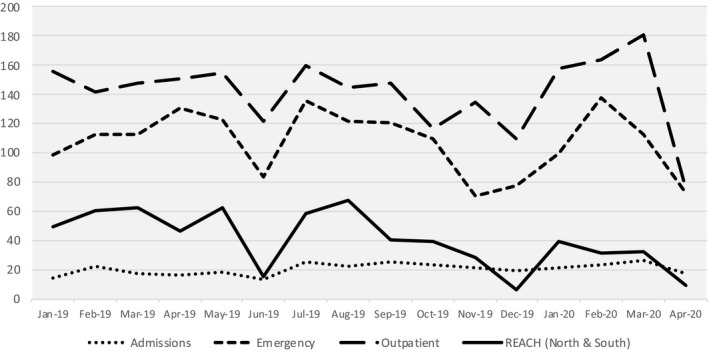
New Referrals across Child and Adolescent Mental Health Services at Singapore’s Institute of Mental Health between January 2019 and April 2020. Note: The figure shows new referrals increased across services in February 2020 (the height of the pandemic) and declined when the stay‐at‐home order/circuit‐breaker period was instated in April 2020

Initially, face‐to‐face community and home visits were postponed to minimize exposure to COVID‐19 for both clients and clinicians. Majority of face‐to‐face services moved to telehealth via videoconferencing or telephone. The novelty of and lack of familiarity with telehealth entailed initial logistical and organizational issues. While telehealth may be unsuitable for some clients, it has shown to be advantageous, such as increasing access to care and patient engagement/satisfaction, and healthcare cost‐effectiveness (Pesämaa et al., 2004). Furthermore, REACH teams worked in split‐team mode and on a two‐week rotation between working from the hospital and home till the ‘circuit‐breaker’ period. Currently, all REACH clinicians work from home and predominantly utilize telehealth and see cases that are difficult‐to‐engage or high‐risk cases in the community. With schools back in session, an increased number of students are reported to have difficulties coping and adjusting back to school. At REACH, there was an increase seen in consultations and referrals. In turn, this has resulted in an increased wait time for interventions of about 2–3 months, whereas the typical wait time is about 1–2 months. Child and adolescent mental health service providers and social welfare agencies also report similar difficulties in meeting the present demands of the population. Further consideration needs to be afforded to increase capacity of the school and mental health services to support youth, in view of the psycho‐social challenges brought upon by pandemic such as COVID‐19. This may include promotion and prevention efforts, such as strengthening mental health literacy, psychological first‐aid training, building resilience and peer‐support programmes. Telehealth may continue to be beneficial after the pandemic ends.

In summary, the COVID‐19 pandemic is causing great disruptions and leading to innovations in education and healthcare sectors. Technology has played a major role during this pandemic. Consideration of asynchronous mental health supports (e.g. apps, artificial intelligence bots, online self‐help) and synchronous support (e.g. helplines, web chat and crisis care) should be further tapped on. While much of the focus of the current pandemic is on the medical management of the virus, the focus needs to turn to the impact of this global health crisis on the mental well‐being of youth during their growing and formative years.

## Ethical information

No ethical approval was required for this article.
